# Progress in Surgery of the Intestines

**Published:** 1903-02-21

**Authors:** 


### Progress in Surgery of the Intestines.

**Intestinal Suture.**—Martin [1] considering the use of the different devices for facilitating sewing, says that the Murphy button and the O'Hara forceps are the most practicable. By the O'Hara forceps the junction is a little more rapid. It offers an admirable means of rapid, easy, safe suture, provided the stitches be passed deeply; but the diaphragm is left too large. The line of the diaphragm is perhaps a third of an inch in width all round, and this may produce sufficient narrowing of the lumen to make a difference between life and death to a patient whose bowels previously have not been cleared out. Also where the bowel walls are thick, the forceps may knuckle or double over. The Lembert suture is fairly rapid, provided the gut be anchored properly. After first closing the mesenteric junction—which should always be done by a rectangular suture tied on the mucous surface—three anchoring sutures are applied, one to either side of the mesenteric attachment and one at the portion of the gut farthest from this attachment. Two continuous sutures are then run from the stitch at the side of the mesentery to that on the convex border. There are then six interruptions. None the less in dilating the bowel there is always a more distinct constriction than where interrupted sutures are placed throughout. The latter, however, take much more time in their application. In regard to all intestinal sutures, it seems clear that the dread of penetrating the mucosa is one of the legacies left from pre-antiseptic days. Cases of fatal peritonitis are not due to penetration of the mucous coat, but to failure to include the sub-mucosa. If the sutures are applied properly, some of them are almost certain to penetrate some part of the mucous membrane. Knots on the outside are supposed to increase the danger of peritonitis. Connell and Maunsell devised their methods of suture for the purpose of avoiding this danger. Connell's suture has seemed strong, perfectly safe, and has produced the smallest diaphragm. The Maunsell method is rapid, easy, and safe. The main objection to it is that it requires an added incision, which must be closed by the Lembert suture. MacLaren [2] says that his experience goes to prove that the method of suture proposed by Connell gives better results, both immediate and permanent, than the Lembert suture, and can be placed almost as quickly as the button.

**Tumours of the Mesentery.**—Moynihan [3] divides tumours of the mesentery into the cystic and solid. A. Mesenteric cysts. The following classification includes all the forms of cystic disease met with in the mesentery:—(1) Dermoid cyst; (2) chylous; (3) sanguineous; (4) serous; (5) hydatid; (6) cysts of adjoining organs encroaching on the mesentery; (7) cysts with walls which structurally resemble intestine; (8) cystic malignant disease. Sanguineous cysts are almost always traumatic. The origin of serous cysts of the mesentery from hæmorrhage has been especially insisted upon by Richet. A hæmorrhage into the mesentery then may result in:—(*a*) A pure blood cyst; (*b*) a sero-sanguineous cyst—serous cyst; (*c*) a hæmorrhage into the pre-existing cyst; (*d*) a hæmorrhage into a mesenteric growth; (*e*) a solid tumour. The most characteristic signs of mesenteric cysts are briefly:—(1) Prominence of the fluctuating tumour towards the umbilicus; (2) great mobility, especially in the transverse direction, and the possibility of rotation round a central axis; (3) the presence of a zone of resonance around and a belt of resonance across the cyst. The symptoms are but little aggressive. A gradually enlarging tumour is observed, but is painless, involves neither suffering nor discomfort, and does not in any way attract attention save by its bulk. Aspiration, incision and drainage, and complete excision have all been practised. Incision and drainage in the recorded cases has a mortality of only 5 per cent. Enucleation of the cyst has a recorded mortality of 60 per cent., but in many cases the operation was performed for acute intestinal obstruction dependent upon the mechanical conditions resulting from an abdominal tumour. Enucleation is, however, the ideal procedure, and should be adopted in most cases. B. Solid tumours of the mesentery. About 30 per cent. of all mesenteric tumours are solid. They are of the following varieties:—(1) Epithelial and endothelial growths (carcinoma and endothelioma); (2) connective tissue growths (fibroma, lipoma, sarcoma); (3) lymphoid growths (lymphoma); (4) vascular growths (angioma, lymphangioma); (5) nerve tissue growths (neuroma). Every form of solid tumour is liable to secondary changes, chondrification, caseation, ossification, etc. The portion of the mesentery most frequently affected is that which is attached to the lower portion of the ileum. A small tumour growing on the posterior part of the mysentery may implicate the vessels of a large segment of the intestine. The posterior attachment of the mesentery is never more than about eight inches, the intestine to which it is carrying blood-vessels is about twenty-five feet in length. The gravity of an operation depends therefore upon the extent of the intestine which has to be removed with the tumour.

**Enteroptosis and Splanchnoptosis.**—Lambotte [4] says the essential feature of enteroptosis is the detachment and descent of the insertion of the colon at the hepatic and splenic flexures, and an elongation of the mesentery. The stomach is always found to be dilated. The precise seat of the obstruction is at the extremity of the duodenum, where the superior mesenteric artery and vein, with the nerve-cords and fat around them, cross the front of the third portion, and owing to the traction exerted on them by the displaced intestine, tend to compress the underlying gut. As a result the duodenum is found dilated, especially in its third portion. Nephroptosis is very frequently associated. The elongation and increased curvature of the colon are prejudicial to the progress of the fæces, and scybalous masses are very commonly met with, which excite, by their presence, irregular and painful peristaltic contractions. This form of "enteralgia" is a prominent symptom of Glenard's disease, as is also the passage of "muco-membranous" stools, similarly due to the irritation of these scybala. Gastric colic, pyloric spasm, dyspepsia, and hyperchlorhydria are all secondary to the duodenal obstruction. The operation practised by the author upon his cases is as follows:—A medium incision is made, partly above and partly below the umbilicus, passing to its left side. It is necessary to decide upon the sites where the new hepatic and splenic flexures must be formed, and these being chosen it remains to fix the gut firmly to the abdominal wall at these places. This is done by attaching ligatures to the bowel at suitable points, and the ligatures are then brought out through the abdominal wall by passing a handled needle from without inwards through the parietes at the points chosen for fixation. The threads are tied over a roll of gauze. The abdomen is closed without drainage and the sutures are removed about the ninth or tenth day. Robinson[5] says there are three stages of splanchnoptosis that can be recognised: (1) Relaxation of the abdominal wall; (2) splanchnoptosis; (3) gastro-duodenal dilatation. In moderate degree of splanchnoptosis, the author has found considerable relief to follow the use of an inflatable, wedge-shaped rubber air-pad, worn inside a binder. The operation advised to strengthen the abdominal wall is that of splitting the sheaths of the recti-abdominales, and enclosing both the recti in one sheath by uniting the sheaths behind and in front of the muscles. This may be performed without opening the peritoneal cavity. In all advanced cases gastro-duodenal dilatation exists, and this is best overcome by performing a gastro-jejunostomy.

**Enterectomy.**—The removal of large portions of the intestines, with recovery of the patient, is becoming much more common than in former years. Harris[6] reports a successful case in which nearly 8 feet of gangrenous intestine were successfully removed. Dowd[7] reports a successful case of resection of a gangrenous intersusception in a child of four years. Drew[8] records a case of artificial anus following strangulation of a ventral hernia successfully treated by resection of small intestine and implantation of the ileum into the cæcum; and Joyat[9] a successful resection of 22 inches of injured bowel with end-to-end anastomosis by means of a Murphy's button.

**Intestinal Obstruction.**—Delatour[10] formulates the following conclusions:—(1) The sudden onset of obstruction of the bowels does not always mean the presence of a recent lesion, especially in those at or beyond 40 years of age. (2) In many cases the symptoms may point to an attack of appendicitis, as the appendix becomes distended by gas or fæcal matter. (3) It is best in all cases where a new growth is suspected, or where there is much distension, to first make an artificial anus, and to use the cæcum for this. At this time do not spend valuable time, and produce some shock, by making a thorough exploration of the entire abdomen, in order to satisfy yourself of the exact conditions. (4) Always open the intestine immediately, as you need to give relief as soon as possible, and there is no danger of leakage of fæces back into the abdomen, provided the exit at the anus is not blocked by too tight dressings. Better leave the wound exposed, the nurse being instructed to clean away the fæcal matter as it appears. (5) Do not be content to leave these cases with the artificial opening, unless at the primary operation the tumour has been found immovable and anastomosis impossible, for many cases may live months with the tumour *in situ* if the current of fæcal matter is diverted by placing the loop of intestine containing it out of the fæcal current. Lockwood[11] classifies the causes of intestinal obstruction into—(1) mechanical, (2) inflammatory, (3) paralytic, (4) vascular. After the failure to get relief by the administration of one or two enemata, laparotomy should be performed.

[1] Annals of Surgery, June 1902, p. 820. [2] Ibid., May 1902, p. 610. [3] Med. Chron., Sept. 1902, p 345. [4] Ibid., March 1902, p. 440. [5] Ibid., p. 442. [6] Med. Rec., Oct. 11, 1902, p. 563. [7] Ibid., Aug. 2, 1902, p. 188. [8] Brit. Med. Jour., June 7, 1902, p. 1402. [9] Lancet, May 31, 1902, p. 1540. [10] Med. Rec., Oct. 18, 1902, p. 610.

